# Brain inspired iontronic fluidic memristive and memcapacitive device for self-powered electronics

**DOI:** 10.1038/s41378-025-00882-x

**Published:** 2025-02-28

**Authors:** Muhammad Umair Khan, Bilal Hassan, Anas Alazzam, Shimaa Eissa, Baker Mohammad

**Affiliations:** 1 Center for Cyber-Physical Systems - System on Chip Lab, Khalifa University, Abu Dhabi, 127788 UAE; 2 Department of Computer and Information Engineering, Khalifa University, Abu Dhabi, 127788 UAE; 3 Department of Electrical Engineering, Khalifa University, Abu Dhabi, 127788 UAE; 4 Division of Engineering, New York University, Abu Dhabi, 129188 UAE; 5 Department of Mechanical and Nuclear Engineering, Khalifa University, Abu Dhabi, 127788 UAE; 6 Department of Chemistry, Khalifa University, Abu Dhabi, 127788 UAE

**Keywords:** Electrical and electronic engineering, Chemistry, Electronic properties and materials, Nanofluidics

## Abstract

Ionic fluidic devices are gaining interest due to their role in enabling self-powered neuromorphic computing systems. In this study, we present an approach that integrates an iontronic fluidic memristive (IFM) device with low input impedance and a triboelectric nanogenerator (TENG) based on ferrofluid (FF), which has high input impedance. By incorporating contact separation electromagnetic (EMG) signals with low input impedance into our FF TENG device, we enhance the FF TENG’s performance by increasing energy harvesting, thereby enabling the autonomous powering of IFM devices for self-powered computing. Further, replicating neuronal activities using artificial iontronic fluidic systems is key to advancing neuromorphic computing. These fluidic devices, composed of soft-matter materials, dynamically adjust their conductance by altering the solution interface. We developed voltage-controlled memristor and memcapacitor memory in polydimethylsiloxane (PDMS) structures, utilising a fluidic interface of FF and polyacrylic acid partial sodium salt (PAA Na^+^). The confined ion interactions in this system induce hysteresis in ion transport across various frequencies, resulting in significant ion memory effects. Our IFM successfully replicates diverse electric pulse patterns, making it highly suitable for neuromorphic computing. Furthermore, our system demonstrates synapse-like learning functions, storing and retrieving short-term (STM) and long-term memory (LTM). The fluidic memristor exhibits dynamic synapse-like features, making it a promising candidate for the hardware implementation of neural networks. FF TENG/EMG device adaptability and seamless integration with biological systems enable the development of advanced neuromorphic devices using iontronic fluidic materials, further enhanced by intricate chemical designs for self-powered electronics.

## Introduction

**Fig. 1:**
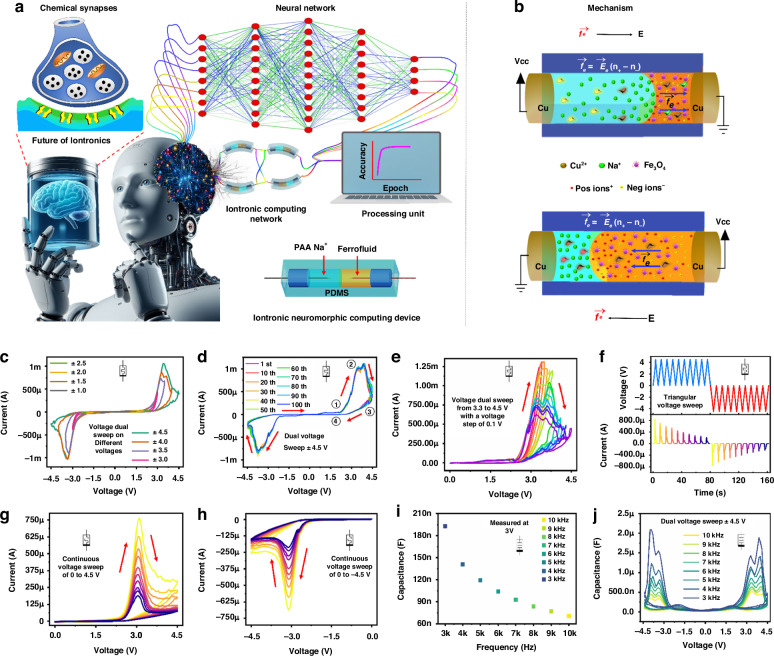
Neuromorphic Behavior of Iontronic Fluidic Memristor. **a** Illustrating the synaptic response between the presynaptic and postsynaptic neurons compared to the ionic memristor interface with future technology. **b** The primary mechanism schematic manipulates ion and liquid transport in the ionic liquid discrete channel. **c** The device voltage sweeps at different voltages. **d** The device voltage sweep of ±4.5 V for 100 endurance cycles. **e** The step response from 3.3 to 4.5 V with a voltage step of 0.1 V. **f** The current and voltage response with time domain for continuous positive and negative voltage cycles. The multi-state switching behaviour at dual voltage sweep of **g** 0 V to 4.5 V to 0 V and **h** 0 V to −4.5 V to 0 V. **i** The capacitance response of the device at a bias voltage of 3 V in a frequency range of 3–10 kHz. **j** Capacitive voltage sweep at ±4.5 V of ionic device in a frequency range of 3–10 kHz

Neuromorphic computing systems aim to achieve the same density, connectedness, and efficiency as the brain^1–6^. To do this, networks of highly parallelised dynamic elements and materials with signal processing and memory capabilities are located in the same place, similar to biological synapses^1,7^. The biological synapse is the crucial connection for signal processing in brain-like networks^2,7^. The ion–neurotransmitter–ion route at chemical synapses plays a significant role in the intricate neurological processes observed in advanced organisms such as humans^8–10^. Comprehending and replicating chemical synapses operational processes is crucial to implementing synaptic intronic devices successfully^11^. Chemical synapses have three main elements: the presynaptic neuron, the synaptic cleft, and the postsynaptic neuron^8,10^. During a synaptic event, an electrical ion current is transmitted from the presynaptic neuron^8,12^. This current triggers the release of neurotransmitters into the synaptic cleft, which in turn stimulates the postsynaptic neuron^12^. As a result, the signal is transmitted from the presynaptic neuron to the postsynaptic neuron^9,10^ (as depicted in Fig. 1a).

Despite this, solid-state devices simulating the electric pulse pattern are utilised for most neuromorphic functions executed thus far. The simulation of a chemical synapse in a solution-based environment uses these solid-state devices^13–15^. In an aqueous environment, fluidic-based memristors^16^ are regarded as the most advantageous devices for achieving neuromorphic functions due to their remarkable compatibility with biological systems and capacity to imbue neuromorphic devices with an extensive array of capabilities via the incorporation of various chemistries^17^. To optimise the performance of neuromorphic computing by simulating synaptic behaviours in neuronal networks via synaptic iontronic devices, it is vital to have a thorough understanding of the synaptic properties and the process by which the devices replicated these properties^18^ (Fig. 1a). Ion solutions permit the coexistence and navigation of various ion types^19^.

Furthermore, ions function as substantial carriers of charge in biological systems as well as fluidic memristors^20^. Therefore, the communication between devices and actual neurons is facilitated by the unparalleled convenience that the fluidic memristor’s biological compatibility affords^21^. Moreover, by incorporating an ionic liquid electrolyte interface into discrete channels, continuous plasticity was successfully attained^22^. Several studies have reported the presence of memcapacitance in confined systems. While a few fluidic memristors have been developed, there’s been limited progress in physically realising memory capacitors. Currently, there’s no realisation of devices exhibiting ideal analogue memcapacitance resulting from molecular-scale geometrical changes in materials^17,18,21–24^. It is critical and highly desirable to conduct independent research on memcapacitive based on iontronic fluidic materials. Exploring alternative memcapacitive materials to overcome these challenges is crucial and highly desirable. Additionally, most observed memcapacitive behaviours are linked with memristive switching, with little attention paid to the memcapacitive mechanism itself^24–26^. This lack of focus undermines the rational and effective design and optimisation of memcapacitors in the future. The field of iontronic offers an unprecedented prospect for developing neuro iontronics devices based on memristor and memcapacitor behaviour that emulate the operational capability of the human brain^1,16,25^.

As the development of advanced human-machine interfaces (HMIs) continues to accelerate, there is an increasing demand for devices that are not only energy-efficient but also capable of performing multiple functions, such as sensing, memory storage, and learning^8,27^. TENGs are particularly valuable in this context due to their versatility, especially when integrated with synaptic resistive memory devices^27^. These memory devices mimic the behaviour of biological synapses, thereby enabling both memory retention and learning capabilities within the system^27^. By merging the sensing and energy-harvesting functions of TENGs with the memory and learning functionalities of synaptic resistive memory devices, it is possible to create intelligent neuromorphic systems that operate with remarkably low power consumption^27^. TENGs, when integrated with these memory devices, can generate spike-like electrical impulses that closely resemble the action potentials found in biological neural networks^27^. Previous approaches to integrating TENGs with synaptic resistive memory devices have largely focused on systems with high input impedance^27–29^. TENGs, naturally possessing high input impedance, are well-suited for powering resistive memories with high input impedance characteristics. However, a significant challenge remains that there is currently no effective method for self-powering low input impedance iontronic fluidic memristor (IFM) devices using TENG/EMG.

The incorporation of ferrofluid (FF) presents a novel hybrid solution. In this approach, the TENG’s active layer is engineered to be sensitive to magnetic fields, which enhances the overall performance of the TENG. Additionally, using an electromagnetic coil provides a hybrid approach which reduces the input impedance, thereby enabling the efficient powering of IFM. This innovative combination improves FF TENG/EMG based systems functionality and expands their applicability in next-generation IFM neuromorphic devices for self-powered electronics as compared in Supplementary Tables S1 and S2. This paper presents an IFM capable of effectively performing various neuromorphic activities. These capabilities include emulating electric pulse patterns and chemical-electric signal transduction. IFM is motivated by biological ion channels, which perform the job of natural memristors by regulating ion flow via spatial confinement. Using a fluidic interface consisting of FF and PAA Na^+^, we developed and manufactured a PDMS fluidic channel iontronic memory, as shown in Fig. 1a. The mechanism is based on ion migration, electroosmosis, and redox reactions. The ability of iontronic devices to precisely regulate the movement of ions in a fluidic medium enables the creation of memory systems with history-dependent behaviour, mimicking biological synapses. The fluid was contained by PAA Na^+^ in this manner, which allowed for the construction of cation concentration equilibrium and charge balance between the inside and outside of the fluidic medium. This occurred under the stimulation of electric fields, causing a redox reaction that injected Cu^2+^ ions, ultimately leading to the formation of history-dependent ion memory. The apparatus demonstrates properties typical of biological synapses, such as spike rate-dependent plasticity (SRDP). In addition, the device has features that characterise memory functions in the brain, such as long-term memory (LTM) and short-term memory (STM). Changes to the weights of the IMF are very reproducible and have been successfully examined for the creation of a convolutional neural network (CNN) with reasonable accuracy in output. Furthermore, by integration of the FF TENG/EMG energy-harvesting system with IFM devices to create intelligent systems that mimic the complex signal transduction and learning behaviours of the brain, offering substantial progress toward energy-efficient, bio-compatible, and high-performance neuromorphic computing platforms. Furthermore, the flexibility and bio-compatibility of IFM devices make them ideal for soft robotics applications, where their ability to mimic synaptic functions can enable adaptive control, sensory feedback, and real-time learning in soft robotic systems, enhancing their responsiveness and versatility in dynamic environments.

## Results and discussion

### Mechanism of IFM

Iontronic devices precisely regulate the movement of cations and anions^16,30,31^. The fundamental operational concept of iontronic devices depends on the ability to alter their electrical characteristics selectively. The behaviour of electrolytes is the fundamental basis of the physics that enables the existence of fluidic memristors^10^. As illustrated in Fig. 1b, when a voltage signal is applied across a fluidic channel, even a small voltage can generate an enormous electric field ${\vec{E}}_{e}$, which induces the cations to drift along the channel. This liquid motion in the presence of an electric field ${\vec{E}}_{e}$, is known as electroosmosis. Prolonged application of the sweep voltage would sustain the migration and motion of ions, thereby potentially introducing substantial non-linearities into ionic transport. The fundamental mechanism for discrete channel devices is based on ion migration, concentration polarisation, and redox reactions (Cu^2+^ ions form through anodic oxidation at the positive electrode, while Na^+^ ions from the polyacrylic acid migrate toward the negative electrode). In this device, ions with identical surface charges repel one another and are attracted to ions with opposite charges, as described by the electroosmotic flow equation in Fig. 1b. Notably, because the ion selectivity on the FF side is negligible compared to the PAA Na^+^ solution, the electric body force $\vec{{f}_{e}}$ is only considered on the PAA Na^+^ side. In contrast to the ions in the bulk region of FF, the predominant factor in transport is the counterions (positive and negative ions) that are attracted to the charges on the wall surface of PAA Na^+^. During the presence of an applied electric field ${\vec{E}}_{e}$ on an interface between FF and PAA Na^+^, the electric body force $\vec{{f}_{e}}$ generates an imbalance between co-ions and countries $({n}_{+}-{n}_{-})$, resulting in a net charge. This phenomenon is illustrated in Fig. 1b. The conductance tuning in our discrete channel device will enable neuromorphic computing to be executed due to the electroosmotic flow. It is possible to see a decrease in conductance during electrical body force due to electrode metallisation resulting from the oxidation and reduction reactions. Cu^2+^ ions mobility plays a significant part during electroosmotic flow, leading to ion concentration polarisation and electrode metallisation. As a result of the diffusion of concentration gradient flux, the flow of ions reduces, ultimately leading to the formation of a state with high resistance (metallisation process). After the polarity of the voltage is altered, the Cu^2+^ ions will travel in the opposite direction, resulting in the conduction of ions becoming weak. During each voltage sweep, this process will be repeated, and the end outcome will be a reduction in conductance due to electrode metallisation.

### Memristor and memcapacitor

Solid-state memristor’s energy consumption and functionality are still not on par with biological neurons that use ions as information carriers^5^. Thus, creating artificial memristors that use ions as information carriers opens new research avenues for scientists to pursue. Among the ionic-based memristors, IFM memristors have advanced quickly in recent years^3,32,33^. Electronic synapses rely on electrons and holes for conduction, whereas biological synapses primarily use ions for information transit and processing. This is the primary difference between conventional synapses and their counterparts^34,35^. Ions have recently shown promise as a candidate for use in identifying synaptic devices due to ion mobility being significantly lower than electron mobility^31,36^. Due to their diverse structures, ionic current can provide more information than electronic current because ions have various valences, sizes, and polarizabilities. Figure 1c shows the measured current–voltage (*I*–*V*) sweep characteristics on different voltage sweeps from ±1 V to ±4.5 V with a voltage step sweep of 0.5 V, which reveals that hysteresis and conductance tuning are visible with only PAA Na^+^ and FF in the discrete channel of IFM. Based on Fig. 1d, we may deduce that when the voltage is positive, an electrical body force will be generated inside the PAA Na^+^ solution and directed from that side of the device to the FF side. The PAA Na^+^ solution is pushed against the FF by this force, and the fluidic channel’s total conductance is improved. This *I*–*V* explains (Fig. 1d) the initially accelerated increase in electroosmotic flow caused by the movement of metallic ions Cu^2+^ under applied voltages, as indicated by the increasing slopes of the marked region ①. The device will undergo a high resistance state after a specific voltage due to the ion concentration of polarisation at the anode and cathode marked region ②. This stage of high resistance will increase as a marked region ③. The electrical current drops following the reverse biased voltage, shown by marked region ④. The same procedure will be used for the negative voltage sweep.

Starting from an initial voltage sweep in the positive direction. The voltage reaches a sufficient positive value to induce an anodic current, causing oxidation of the copper metallic electrode and forming Cu^2+^ cations, which subsequently migrate to the negative electrode. The redox reaction persists until most surfaces of the electrode are oxidised, peaking the anodic current. Subsequently, the current decreases for the remaining forward scan duration until the voltage sweep is reversed. The current decreases until the potential reaches a point where the reduction of Cu^2+^ begins, resulting in the formation of Cu metal. The cathodic current peaks at this point, coinciding with a notable reduction in Cu^2+^ ion concentration. The cathodic current then decreases from its peak after the ion concentration of polarisation. The negative sweep proceeds like the positive scan.

Figure 1e demonstrates the effects of raising the SET-stop voltage from 3.3 V to 4.5 V. It was observed that the SET-stop voltage influences the resistance values in both the LRS and HRS. Notably, multiple switching states can be achieved by incrementally increasing the SET voltage by 0.1 V. To confirm the conductance and shift even further, we performed ten successive triangular positive (+4 V) and negative (−4 V) voltage sweeps with time, as seen in Fig. 1f. For successive positive and negative sweeps, the findings indicated a reduction in the current of the device over time; these features are seen as most advantageous for device use in neuromorphic computing. Setting or reset pulses may restore this adjustable state to its starting point. These results imply that memristor conductance may be gradually adjusted by applying optimum programming voltage pulses on the devices’ presynaptic terminals. Figure 1g, h depict the memristive characteristics of IFM, captured through a series of voltage sweeps. Initially, ten consecutive sweeps were applied from 0 V to +4.5 V to 0 V, followed by ten consecutive sweeps from 0 V to −4.5 V to 0 V. It is noteworthy that the conductance of the device decreased progressively with each sweep, regardless of whether a positive or negative voltage was applied. A particularly interesting observation is that the second sweep almost entirely overlaps with the first, highlighting a distinct change between successive sweeps.

Capacitive neural networks offer an alternative physical implementation of resistive-based neural networks that more accurately emulates neural functions and potentially reduces circuit power dissipation due to the conversion of signals from current to voltage^23,24,37–39^. As a result, the memcapacitive characteristics of IFM devices at the intronic fluid interface of FF and PAA Na^+^ are investigated. As the frequency increases from 3 kHz to 10 kHz, the capacitance decreases progressively in the capacitance–frequency (C–F) measurement with a bias voltage of 3 V (Fig. 1i). Due to the limited time available for ions to traverse the electrical field and generate an electrical double-layer capacitance at high frequencies, high-frequency capacitance is diminished compared to low-frequency capacitance. As frequency increases from 3 kHz to 10 kHz, capacitances decrease from 172.98 to 70.80 nF in the C–F curves. As the biassing frequency increases from 3 kHz to 10 kHz, the capacitance amplitude and the hysteresis loop areas decrease, as illustrated in Fig. 1j. Using memory characteristics, the device can convert between the low and high capacitance states (LCS and HCS). The capacitance of the ON state is significantly greater than that of the OFF state in our experiment. The decrease in capacitance of the HCS with increasing frequency is attributed to the greater likelihood that positive charge (Na^+^ and Cu^2+^) becomes confined at its interface with the electrode at lower frequencies. Therefore, the optimal frequency should be selected to maximise the capacitive difference between the two phases.

### Neuromorphic computing

**Fig. 2:**
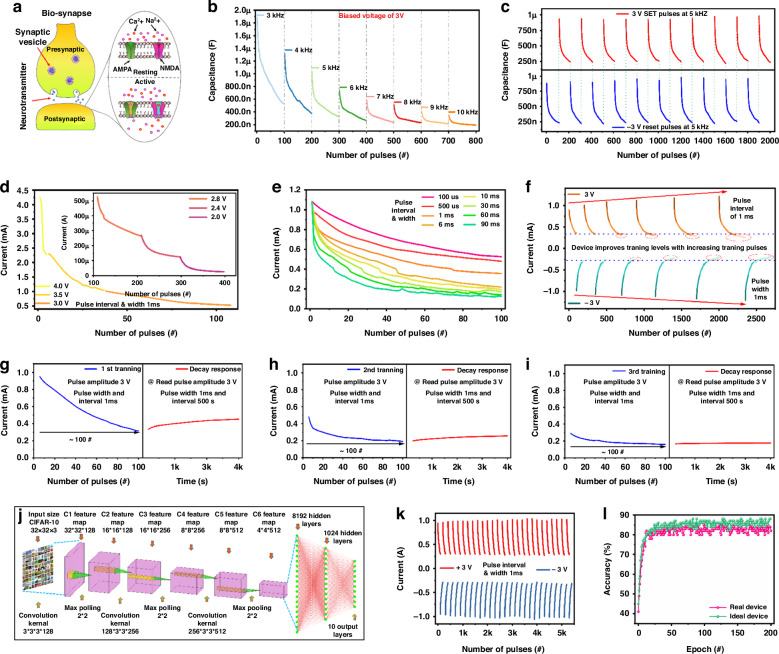
Synaptic Behavior and Training Characteristics of Iontronic Fluidic Memristor. **a** Illustration of the biosynapse similar to IFM behaviour. **b** The capacitance spiking response was measured in a frequency range of 3 kHz to 10 kHz with a pulse width of 1 ms. **c** The stability response of capacitance behaviour at 5 kHz with positive +3 V set and −3 V reset pulses with a pulse width of 1 ms. **d** The different pulse amplitude responses at 2.0, 2.4, 2.8, 3, 3.5, and 4 V with u;lse 1 ms and pulse interval 1 ms. **e** Plasticity characteristics with varying pulse intervals and intervals of 100 μs, 500 μs, 1 ms, 6 ms, 10 ms, 30 ms, 60 ms, and 90 ms. **f** The device training will increase by 50 pulses for each set and reset the level of training of iontronic devices. The transition of the IFM device from STM to LTM was evaluated through three distinct phases: **g** the first learning and decay process, **h** the second learning and decay process, and **i** the third learning and decay process. **j** Neurosim 2.0 convolution Neural network based on GCC 8. **k** The device retention and endurance test for 5400 pulses with stable set and reset process. **l** The output accuracy of CIFAR-10 is based on the weight update information of IFM

Promising synaptic characteristics of iontronic fluidic devices have been shown in the electrochemical responses of the ions, which can replicate the movement of ions in the nervous system, so these characteristics can be used to build synaptic devices. Every process in the brain that involves encoding, transferring, or decoding information begins with the neuron (Fig. 1a). In biosynapses, an action potential moving through the presynaptic neuron triggers the opening of voltage-gated calcium (Ca^2+^) channels. This leads to an influx of Ca^2+^ ions into the presynaptic terminal. The Ca^2+^ ions then bind to synaptic vesicles loaded with neurotransmitters, facilitating their docking at the presynaptic plasma membrane. Once docked, these vesicles fuse with the membrane, releasing neurotransmitters into the synaptic cleft, the space between the presynaptic and postsynaptic neurons. The released neurotransmitters then bind to specific receptors on the postsynaptic plasma membrane, particularly to the α-amino-3-hydroxy-5-methyl-4-isoxazolepropionic acid (AMPA) and N-methyl-D-aspartate (NMDA) receptors. These receptors function as ion channels, and their activation by neurotransmitters leads to the opening of these channels, allowing ions to flow into the postsynaptic neuron. This ion influx, primarily involving sodium (Na^+^) and Ca^2+^ ions, causes depolarisation of the postsynaptic cell, a critical step in transmitting the signal to the next neuron in the network. It is important to note that, under resting conditions, these ion channels remain closed, maintaining the postsynaptic membrane in an insulating state. This insulating state is disrupted only when neurotransmitters bind to their respective receptors. Upon binding, the conductance of the postsynaptic membrane increases sharply, enabling the ion flow that drives neuronal communication. This precise mechanism ensures that signals are transmitted efficiently and only in response to specific neurotransmitter binding, maintaining the fidelity of neural communication. Spiking signals are how neurons communicate, as shown in Fig. 2a. The relationship between frequency and capacitance in memcapacitance devices is crucial for understanding their behaviour in neuromorphic computing. In our memcapacitance spiking test, we performed capacitor-based neuromorphic computing across a frequency range of 3–10 kHz, with a frequency step of 1 kHz, applying 100 pulses during each test with a pulse width of 1 ms as shown in Fig. 2b. During this frequency sweep, a clear trend of decreasing capacitance was observed. At 3 kHz, the capacitance dropped significantly from 1.88 µF to 532 nF. As the frequency increased to 4 kHz, it decreased from 1.31 µF to 383 nF. This trend continued at 5 kHz, with the capacitance falling from 1 µF to 332 nF, and at 6 kHz, it was reduced from 742 nF to 273 nF. Similar reductions were observed at 7 kHz (from 584 nF to 238 nF), 8 kHz (from 509 nF to 231 nF), 9 kHz (from 409 nF to 216 nF), and finally at 10 kHz, where it dropped from 332 nF to 195 nF. This progressive reduction in capacitance as the frequency increases suggests that higher frequencies compress the range of capacitance variation, likely due to reduced time for charge redistribution within the memcapacitance device. Consequently, the device’s response becomes increasingly nonlinear, posing challenges for stable neuromorphic computing as its behaviour becomes less predictable. This study provides valuable insights for selecting an optimal frequency range that offers a stable response with a more pronounced difference between maximum and minimum capacitance values and a more linear synaptic change. Such an approach is essential for the effective hardware implementation of neural networks, enabling more reliable and consistent performance in neuromorphic systems, which is crucial for developing efficient and scalable hardware for artificial intelligence applications. Based on the frequency test (3 kHz to 10 kHz), we selected 5 kHz to evaluate the stability of the capacitance spiking response during the set and reset processes, as shown in Fig. 2c. At 5 kHz frequency, 100 pulses were applied for both the set and reset operations, with voltages of +3 V and −3 V, respectively, and a pulse width of 1 ms. The choice of 5 kHz was selected by its more stable capacitance response, where the capacitance decreased from 983 nF to 241 nF during the set process and from 978 nF to 237 nF during the reset process. This stability indicates that 5 kHz is an optimal frequency for achieving consistent and reliable performance in capacitance-based neuromorphic computing, as it offers a more predictable and uniform response during both operational phases.

These signals of IFM can be distinguished by pulse and frequency-modulated nature, referred to as spike rate-dependent plasticity (SRDP). The pulse duty cycle and amplitude were varied to investigate the IFM device’s SRDP in the discrete channel. As shown in Fig. 2d, the current drops from 4.26 mA to 25.8 µA at various voltage amplitudes of 2.0, 2.4, 2.8, 3.0, 3.5, and 4 V with a pulse width of 1 ms and an interval of 1 ms. This indicates a lower electrical body force between the interface of FF and PAA Na^+^ at a lower voltage than a higher voltage. As seen in Fig. 2e, the synaptic weight can be controlled by sequential stimuli of externally applied pulses of varying widths 100 μs, 500 μs, 1 ms, 6 ms, 10 ms, 30 ms, 60 ms, and 90 ms and intervals of 100 μs, 500 μs, 1 ms, 6 ms, 10 ms, 30 ms, 60 ms, and 90 ms corresponds to frequency response (1/(pulse interval + pulse width) in a range of 5 kHz, 1 kHz, 500 Hz, 83.3 Hz, 50 Hz, 16.7 Hz, 8.33 Hz, and 5.67 Hz, respectively. The 10 μs pulse width does not indicate a noticeable drop in current, maintaining a level from 1.081 mA to 526.86 μA. However, when comparing longer pulse widths of 100 μs, 500 μs, 1 ms, 6 ms, 10 ms, 30 ms, and 60 ms, with 90 ms the current changes significantly from 1.081 mA to 125.19 μA with a high *I*_on/off_ ratio of 8.65. The device has a 90 ms duty interval, which results in significant non-linearity. To achieve high convergence with low non-linearity, a suitable duty cycle is selected of 1 ms with a current change from 1.081 mA to 356.77 μA *I*_on/off_ ratio of 3.03, which provides good non-linearity value of 2.63. It is possible that using these weight change factors would be beneficial to creating an application that utilises convolutional neural networks (CNN) for data reorganisation.

High-frequency operation is crucial for real-time neuromorphic applications, but it introduces several challenges related to the rate of change of current in IFMs. When operating at frequencies such as 5 kHz, the current change rate significantly slows down due to the inability of ions to migrate fast enough within the electric field. The ions require a longer pulse width to migrate and redistribute at this frequency. This extended pulse width reduces the system’s responsiveness, as the ions cannot follow the rapid changes in the applied voltage. This slow reaction time negatively impacts the effectiveness of weight updates during the training of neuromorphic systems, making 5 kHz unsuitable for efficient device operation in these applications. In contrast, at very low frequencies, such as 5.67 Hz, the current change rate is too fast for the device to handle effectively. While the ions may be able to migrate more quickly, the system becomes prone to non-linearity and instability, as the rapid current changes lead to a mismatch between the expected and actual behaviour of the device. The system’s response becomes erratic, and the device fails to maintain consistent performance at such low frequencies, which are unsuitable for real-time neuromorphic tasks. Through our characterisation of the system between 5 kHz and 5.67 Hz, we found that 500 Hz offers the best balance between efficient ion migration and stable device operation. At 500 Hz, the ions can move with sufficient speed to track the applied voltage changes while avoiding the slow response seen at higher frequencies. At 500 Hz, the system also ensures that weight updates occur optimally for training the neuromorphic system. This frequency balances speed and stability, enabling the system to operate effectively without performance degradation at higher frequencies or instability at much lower frequencies. Therefore, 500 Hz is the most suitable frequency for iontronic fluidic memristors in neuromorphic computing, offering the best rate of change of current for real-time applications.

Several strategies can be proposed to alleviate the degradation of device performance at high frequencies. First, optimising the electrode materials can help minimise polarisation effects, significantly hindering ion mobility at high frequencies. This can be achieved by selecting materials that facilitate better ion transport and reduce the accumulation of charges at the electrode-electrolyte interface. Second, improving the ionic conductivity of the electrolyte material can enhance ion migration speed, enabling the device to operate more efficiently at higher frequencies. This can be done by engineering the fluidic properties of the electrolyte, such as reducing its viscosity and increasing its ionic conductivity. Third, using advanced pulse-shaping techniques can help manage the pulse width at high frequencies, allowing for better synchronisation between ion movement and voltage changes, thus improving the rate of change of current. Additionally, integrating optimised ionic liquids with specific characteristics that match the operating frequency range of the device can enhance the system’s overall performance. By refining the frequency range, electrode design, and electrolyte materials, these strategies can help maintain stable performance in iontronic fluidic memristors at higher frequencies, ensuring that they meet the demands of real-time neuromorphic computing applications.

As illustrated in Fig. 2f, the modification procedure facilitated the long-term storage of analogue weights and was completely reversible. This was accomplished by applying one hundred set spikes (+3 V) and reset spikes (−3 V). Linearity is of critical importance for system-level weight tracing. To gain insight into the learning process, a pulse scheme consisting of 100 sets and reset pulses with a duty cycle of 1 ms (pulse width and interval) was devised. For each subsequent set/reset cycle, 50 pulses were added to the previous synaptic training. The device can undergo additional training at higher current levels, and the ion/off ratio will increase with each set of trials. During the training process, as the number of training pulses increases, the device experiences non-linearity, as marked in red circles in Fig. 2f. The weight update behaviour must be nearly linear to ensure efficient tracing and have a favourable *I*_on/off_ ratio. This will facilitate the realisation of neural networks at the hardware level with greater precision.

This work demonstrates that these devices can simulate fundamental functions in biological synapses. Before proceeding, it was verified that the current could be decreased by increasing the voltage of a specific polarity. The conversion from short-term potentiation (STP) to long-term potentiation (LTP) in the device is achieved by modulating its current or conductivity through pulse stimulation, mirroring the human brain’s transformation of STM LTM, as shown in Fig. 2g–i. This process aligns with the Ebbinghaus Forgetting Curve, which describes the cycle of “Learning-Forgetting-Re-learning-Memorising” as the brain assimilates and retains information. The learning behaviour observed in IFMs is attributed to the electrolyte’s ability to preserve its current state by forming stable ion clusters. This process typically occurs within a few milliseconds, enabling the device to emulate memory retention mechanisms effectively. In the initial training phase, as shown in Fig. 2g, the device’s current decreased from 9.5 mA to 0.31 mA after 100 electrical pulses with a 1 ms pulse width and interval. After removing the stimuli, the device was periodically tested every 500 s using a 1 ms pulse width and a 3 V read voltage to measure current decay, which progressed from 0.31 mA to 0.44 mA over 4000 s, mimicking the brain’s short-term learning behaviour. In the second training phase, depicted in Fig. 2h, the device’s current dropped from 0.48 mA to 0.191 mA after another 100 pulses. During the subsequent 4000-s forgetting period, the current changes from 0.191 mA to 0.25 mA. Finally, in the third training phase (Fig. 2i), the current decreased from 0.29 mA to 0.15 mA after 100 pulses, and over the same forgetting period, the current exhibited minimal attenuation, rising only slightly from 0.15 mA to 0.178 mA. This diminished decay signifies the successful transition from STM to LTM, demonstrating the device’s ability to emulate human memory consolidation by retaining information more effectively with repeated training. These findings highlight the potential of such devices for neuromorphic applications by mimicking the human brain’s learning and memory mechanisms.

### Convolutional neural network simulation

Neuromorphic computer systems, which mimic the functions of the human brain, show significant potential for overcoming the limitations of traditional implementation of artificial intelligence algorithms^40,41^. As a biological medium for computation, the brain fundamentally diverges from the conventional architecture of electron-based computing that relies on digital circuits. The brain employs biological elements such as synapses and neurons, whilst the latter depends on memory blocks and transistors. The advent of ion-based computing can potentially transform the existing electron-based system substantially. Ion-based computing offers several advantages, including reduced energy consumption, exceptional compatibility with brain-computer interfaces, and the capacity to employ ions of varying sizes and valence. Fluidic systems have great potential in promoting the development of highly efficient computers, particularly in simulating biological brain networks. For utilising CNN, the current pursuit device array method employs a parallel read-out analogue embedded Non-Volatile Memory (eNVM)-based pseudo-crossbar. Further details can be found in Supplementary Figs. S1 and S2.

The device was used with neuromorphic functionalities, leading to operational data collection. CNN is used to evaluate the proposed IMF. The CIFAR-10 dataset is used as the input data, and six convolutional layers are used to extract features. Subsequently, the last three ultimately linked layers are used to classify these properties, as shown in Fig. 2j. A parallel-read-out analogue eNVM-based pseudo-crossbar detects devices in an array when using CNN. Initially, an endurance test is undertaken to evaluate the device’s weight, which involves subjecting it to 5400 pulses. The device has constant and dependable endurance repeatability over cycles, indicating that the interface between the high-resistance and low-resistance areas stays mostly intact even after removing the set/reset voltages. This value, shown in Fig. 2k, is essential for training on the chip. Furthermore, the device parameters are determined based on the endurance data, encompassing discrete levels, set and reset voltage, maximum and minimum resistance, non-linearity, cycle-to-cycle fluctuation, and duty cycle. The number of pulses shown in Fig. 2k is the same at 100 for both positive (+3 V) and negative (−3 V) stimulation, with a pulse interval of 1 ms and width of 1 ms. In constructing artificial synapses, it is essential to attain a significant degree of consistency in the electrical conductivity of a device when exposed to a series of set/reset voltage pulses. The device’s low resistance is 10.16 kΩ, while its high resistance is 29.70 kΩ. The *R*_off/on_ ratio is around 3.42. The non-linearity coefficient for positive pulse data is 2.74, whereas, for negative pulses, it is 3.13. The cycle-to-cycle variance for positive pulses is 0.032, whereas for negative pulses it is 0.038. Based on this data, kernels and synapses in all layers were trained to reduce the difference between the actual and expected output. Figure 2l illustrates the accuracy of the CNN after 200 epochs, with a recorded output of 85% for the real weights. Further simulation of the ideal device was conducted using a linear function to provide a genuine case comparison. Figure 2l illustrates that the most effective device attains a maximum accuracy of around 87%. The results closely correspond to those obtained from the device in real-life and optimal situations. The low cycle-to-cycle variation in the current IFM, as shown in Fig. 2k, may be attributable to its interfacial memristor nature. Based on device structure and switching voltage, STDP, and CNN accuracy, the performance parameters are compared with already reported work in Supplementary Table S1.

### Self-powered computing of IFM using FF TENG/EMG

**Fig. 3:**
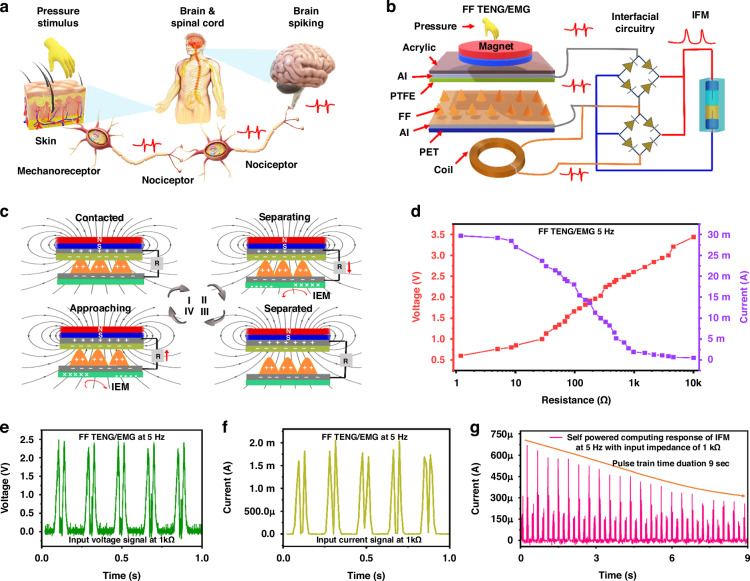
Self-Powered Neuromorphic Computing with Ferrofluid TENG/EMG Integrated with Iontronic Fluidic Memristor. **a** Illustration of the synapse event triggered by the external stimuli. **b** The interface circuitry of FF TEMG/EMG and IFM using a double bridge rectifier shows a self-powered system. **c** Complete working principle of the FF TENG/EMG. **d** The loading resistance effect on current and voltage of FF TENG/EMG. The **e** voltage and **f** current response of FF TENG/EMG at 1 kΩ. **g** Self-powered response of IFM based on the output of FF TENG/EMG at 1 kΩ input impedance

The human body’s biological perception system processes and transmits information through action potentials, which are signals generated by neurons that play a vital role in neural networks. As shown in Fig. 3a, sensory neurons are specialised in detecting stimuli, such as environmental changes or object movements, through bioreceptors. These neurons are essential for processing these signals, ensuring the body responds appropriately to external stimuli. Similarly, neuron-like devices that mimic this function in artificial neural networks must be developed. These devices must generate real-time responses to external stimuli and perform basic computations on the incoming signals, as depicted in Fig. 3b. We have proposed an IFM synaptic device, which processes and stores information and adapts their behaviour through weight adjustments due to synaptic plasticity. This is achieved using the integration of IFM devices with FF TENG/EMG has led to significant advancements in neuromorphic computing, particularly in self-powered systems. This work is compared with self-powered neuromorphic computing resistive memory devices in Supplementary Table S1. This approach eliminates reliance on traditional energy supplies, promoting long-lasting, efficient performance free from conventional power systems’ environmental and operational limitations. These self-powered memory devices allow soft robots to function autonomously without needing bulky, external power sources to power memory computing units. As soft robots demand increasingly sophisticated sensory feedback and adaptive learning, the FF TENG/EMG self-powered IFM system represents a cutting-edge solution, driving forward the development of autonomous, self-powered soft robotics that can be deployed in a wide range of real-world applications. The artificial perception system based on these IFM synaptic devices is advantageous for processing simple, singular data streams. It offers an efficient and responsive model for certain information-processing tasks and the biological systems they are designed to emulate. The FF TEMG/EMG structure is depicted in Fig. 3b. A magnet controls the microstructure of FF by adjusting the magnetic field strength^42^. FF is a liquid containing nanoscale ferromagnetic particles suspended in a carrier fluid, like an organic solvent or water^43,44^, as shown in Supplementary Fig. S3. These particles are coated with surfactants to prevent them from clumping due to van der Waals forces and magnetic interactions. Without a magnetic field, the FF remains non-magnetic. However, when a magnetic field is applied, the ferromagnetic particles align with the field, giving the fluid magnetic properties. In this state, the particles stay well-dispersed, and their arrangement is stable as long as the magnetic field is maintained. When the magnetic field reaches a particular strength, it can overcome the FF surface tension and gravity, leading to normal-field instability, where the fluid’s surface forms folds or peaks due to the interaction between magnetic forces and the fluid’s properties. The FF TENG/EMG working principle (Fig. 3c) shows that PTFE attracts electrons from the FF due to its high electron affinity, resulting in a negatively charged PTFE film and a positively charged ferrofluid (state I). At this stage, no electromagnetic generation occurs since there are no changes in magnetic flux relative to the coil. When pressure is released, positive charges in the FF drive electrons from the negative electrode attached to PTFE (state II). Simultaneously, the EMG initiates an anticlockwise current I_EM_ in the coil due to reduced magnetic flux. With no pressure applied, equilibrium is reached, and no electrical signal is observed in FF TENG/EMG (state III). Upon pressure application, induced electrons flow back to the FF electrode to balance the potential change on the electrode (stage IV). Additionally, a clockwise current *I*_EM_ is induced in the coil, attributed to increased magnetic flux. This cyclic process produces an AC signal with alternating positive and negative pulses. Supplementary Figure S4 illustrates the contact electrification between the FF and PTFE using an electron cloud interaction model. In this model, d represents the distance between electron clouds, E1/2 is the potential energy required for electrons to escape, and EA/B is the energy level of electrons in the material atoms. Before contact, electrons are trapped by potential wells, preventing transfer. Upon contact, electron clouds overlap, forming an asymmetric double-well potential, allowing electron transfer between materials. After separation, the transferred electrons remain due to an energy barrier unless external conditions change.

Pursuing an electronic soft fluidic sensor and memory that emulates the human mechanoreceptor system, enabling communication with nerves and facilitating intelligent sensing, opens new possibilities for self-powered computing applications. Notably, FF-TENG and EMG show significant potential in converting mechanical energy into electrical energy, which is advantageous for neuromorphic computing with IFM devices. However, FF-TENGs, due to their high input impedance, cannot generate the desired output signal at the required input impedance. As illustrated in Supplementary Fig. S5, at a load of 1 kΩ, the system can generate 0.5 V and a current of 30 µA. High impedance can limit the current output, making achieving the desired power levels needed to drive IFM devices difficult. To enhance the performance of the FF-TENG, we introduced an EMG coil with 1200 turns and a coil diameter of 3 cm. The EMG coil, having a low input impedance, can generate a higher current of 1.85 mA and a voltage of 2 V at an input impedance of 1 kΩ, as shown in Supplementary Fig. S6. This improvement is crucial for providing the necessary power to the IFM devices, enabling them to function properly in neuromorphic computing applications. By combining the outputs of the FF-TENG and EMG generators, we can significantly enhance the overall output performance. The combined FF-TENG/EMG generator output is 2.5 V at 1 kΩ with a current of 1.89 mA, as shown in Fig. 3d. The stability of FF TENG/EMG is analysed for 40,000 cycles, as shown in Supplementary Fig. S7. This combination not only improves the output performance of the TENG but also strengthens the input stimuli operation for the IFM. The increased voltage and current ensure that the IFM devices receive a steady and sufficient power supply, which is essential for self-powered computing applications. The output voltage and current signals generated by the FF-TENG/EMG are depicted in Fig. 3e, f, providing detailed insights into how this output signal interacts with the IFM, as shown in Fig. 3b. For computing applications, it is essential to consider pulse width, pulse interval, and pulse potential. The pulse interval is 100 ms, with a rectified pulse width of 58.5 ms, the active pulse width of two rectified pulses being 15 ms, each pulse potential of 2.5 V, a Pulse current of 1.89 mA and a pulse power of 4.725 mW. The output of the IFM, based on these input stimuli, shows a computing current ranging from 668 µA to 257 µA over a 9 s stimulus generated by the FF-TENG/EMG at 5 Hz, as shown in Fig. 3g. This study represents the first detailed investigation into developing a self-powered neuromorphic device using IFM technology. The successful integration of FF-TENG and EMG generators with IFM devices opens new possibilities for creating self-powered systems that can operate independently of external power sources. Such systems could be used in a variety of applications. Self-Powered Soft Skins: These could be used in robotics to create sensors that detect pressure, temperature, and other environmental factors, providing robots with a sense of touch. Soft Robotics: The enhanced performance of IFM devices could lead to the development of advanced soft robots capable of complex, adaptive behaviours.

## Conclusion

This study demonstrated a soft, two-terminal iontronic fluidic memristor and memcapacitor using a fluidic FF and PAA Na^+^ interface within a PDMS channel. The iontronic fluidic memristor (IFM) exhibits neuromorphic behaviours observed in biological systems, making it a promising candidate for advanced neuromorphic computing applications. A key innovation of this work is integrating a self-powered mechanism by combining the iontronic fluidic device with FF TENG/EMG. The TENG, characterised by its high input impedance, is effectively combined with the low input impedance of the IFM device. The contact separation EMG combined with FF TENG improves the ferrofluid TENG’s performance, enabling the IFM device’s autonomous powering. This self-powered capability is particularly significant for the development of neuromorphic systems, where traditional power sources can be bulky, limiting the flexibility and adaptability of these devices. The autonomous energy generation and storage provided by the FF TENG/EMG device eliminates the need for external power sources, enhancing the portability and integration of neuromorphic devices in real-world applications. Furthermore, the self-powered nature of these devices contributes to their long-term sustainability and reliability. By harvesting energy from environmental stimuli, such as mechanical movement or bio-signals, the FF TENG/EMG device can continuously power the IFM, supporting its synapse-like adaptive learning and memory functions, including STM and LTM. This capability not only extends the operational lifetime of the device but also aligns with the principles of energy-efficient and sustainable design, which are increasingly important in modern technological applications. The findings from this work suggest a novel paradigm for neuromorphic hardware that leverages self-powered iontronic fluidic materials to create multifunctional structures. These materials can offer new possibilities for adaptive sensing, signal processing, intelligent edge computing, and memory functions in self-powered robotic and neuromorphic systems. This approach represents a significant advancement in developing flexible, autonomous, and energy-efficient neuromorphic devices that can operate independently and adapt to their environment, making them ideal for a wide range of self-powered wearable sensing applications.

## Materials and methods

Ferrofluid (FF) colloidal dispersion with a 1.21–1.42 g/cc density and a 6–12 cp viscosity was acquired from FerroTec Corporation. The percentages (by volume) of FF colloidal dispersion are as follows: 3-15% iron oxide (Fe_3_O_4_) (also known as magnetite), 3–6% oil soluble dispersant, and 55–91% petroleum distillates. Sigma-Aldrich provided the C_3_H_3_NaO_2_ (PAA Na^+^) polyacrylic acid partial sodium salt solution with a molecular weight of 5000 and a 0.69 g/mL density. Dow Corning provided the PDMS component. As a starting material, hydroxyl-terminated PDMS was mixed with tetraethyl orthosilicate in a 1:10 weight ratio. The mould was assembled using a thin wire with a diameter of 800 µm. The PDMS was hardened at 80 °C for 4 h. The channel had a volume ratio 1:1, with two electrodes serving as anode and cathode. The fabrication process for the combined FF TENG EMG device started with assembling the triboelectric nanogenerator (TENG). Two aluminium (Al) tape electrodes were used, each measuring 4 cm × 4 cm. First, the edges of the Al electrodes were covered with a PDMS mould to secure the ferrofluid (FF) on the surface of one electrode. A thin polytetrafluoroethylene (PTFE) layer was then applied to the other Al electrode, forming the movable part of the TENG and completing its construction. The EMG was then assembled using a 3 cm × 3 cm magnet with a thickness of 1 cm and a coil with a diameter of 3 cm, consisting of 1200 turns of wire with a diameter of 0.12 mm. The coil was attached to the back of the stationary part of the TENG, while the magnet was affixed to the back of the movable part, creating the FF TENG/EMG device. The full device structure is illustrated in Fig. 1a. A linear damping system was designed to operate within a 5 Hz frequency range for performance analysis. The FF TENG/EMG was assessed by measuring the open-circuit voltage with a Rohde & Schwarz RTO 1014 oscilloscope while short-circuiting measurements of FF TENG/EMG and the behaviour of the memristor and memcapacitor in an IFM were conducted using a Keysight 4200A SCS source measurement unit.

## Supplementary information

Supplementary Information
