# Supplementary material for: Brain inspired iontronic fluidic memristive and memcapacitive device for self-powered electronics
Source: Microsyst Nanoeng. 2025 Feb 28;11:37. doi: 10.1038/s41378-025-00882-x (PMC11871289; doi:10.1038/s41378-025-00882-x)
Supplement: Supplementary file 1 — Supplementary Information [file 41378_2025_882_MOESM1_ESM.docx]

**Supporting information:** **Brain Inspired Iontronic Fluidic Memristive and Memcapacitive Device for Self-powered Electronics**

# Muhammad Umair Khan^a,b^, Bilal Hassan^c,d^, Anas Alazzam^a,e^, Shimaa Eissa^f^, Baker Mohammad^a,b^ ^*^

^a^Center for Cyber-Physical Systems - System on Chip Lab, Khalifa University, Abu Dhabi 127788, UAE

^b^Department of Computer and Information Engineering, Khalifa University, Abu Dhabi, 12778, UAE.

^c^Department of Electrical Engineering, Khalifa University, Abu Dhabi, 12778, UAE.

^d^Division of Engineering, New York University, Abu Dhabi, 129188, UAE.

^e^Department of Mechanical and Nuclear Engineering, Khalifa University, Abu Dhabi, 12778, UAE

^f^Department of Chemistry, Khalifa University, Abu Dhabi, 12778, UAE.

Corresponding author: [muhammad.khan@ku.ac.ae](mailto:muhammad.khan@ku.ac.ae); [baker.mohammad@ku.ac.ae](mailto:baker.mohammad@ku.ac.ae)

- **Comparison of liquid neuromorphic resistive memory devices.**
- **Comparison of self-powered resistive memory devices.**
- **CNN simulation architecture used for hardware implementation of the neural network:**
- **Mechanism study and charge transfer study of Ferrofluid TENG.**
- **Power performance of FF TENG and EMG.**
- **Stability of FF TENG/EMG Device**

^*^E-mail: muhammad.khan@ku.ac.ae, [Baker.mohammad@ku.ac.ae](mailto:Baker.mohammad@ku.ac.ae)

| **No** | **Liquid neuromorphic resistive memory Device structure**  **& Device flexibility** | **Device operating voltage, endurance & retention** | **Spiking voltage and number of pulses for potentiation & depression &**  **Spiking pulse width &**  **Weight ratio & spiking Stability** | **Simulation-based on neural network or convolutional neural network** | **Memcapacitance** | **Ref:** |
| --- | --- | --- | --- | --- | --- | --- |
| **1** | Ag/AgCl/KCl/BMIM PF_6_/AG/AgCl  (PDMS substrate)  (Flexiblity test not performedk) | Bipolar resistive swiching  ± 20 V  (50 cycle to cycle endurance) | + 4 V and - 10 V, 1V(read)  (80 pulses for Potentaion and Depression**)**  (0.5 sec pulse width)  Weight ratio ~ 2  Spiking stability 1000 pulses | Convolutional neural network accuracy 97% | No | **^[^**[**^1^**](#_ENREF_1)**^]^** |
| **2** | Cu/Ag@AgCl/Cu  (PDMS substrate) (Flexible) | Multistate resistive swiching  ± 1.5 V  (100 cycle to cycles endurance) | +1.3 V and -1.3 V  (100 pulses for potentiation and depression)  (1 ms pulse width)  Weight ratio ~ 1.5  Spiking stability 400 pulses | Convolutional neural network accuracy 85% | No | ^[^[^2^](#_ENREF_2)^]^ |
| **3** | Cu/Silk Fibron/Cu  (PDMS substrate: highly Flexible and robust)  (Flexible) | Multistate resistive swiching  ± 10 V | +4 V and -4 V  (100 pulses for potentiaon and depression)  (1ms duty interval)  Weight ratio ~ 2.911  Spiking Stability 1300 pulses | Convolutional neural network accuracy 90.16% | No | **^[^**[**^3^**](#_ENREF_3)**^]^** |
| 4 | Cu/BMIM FeCl_4_ : H_2_O/Cu  (PDMS substrate: highly Flexible and robust)  (Flexible) | Multistate resistive swiching  ± 1.5 V | +1.5 V and -1.5 V  (30 pulses for potentiaon and depression)  (1ms pulse width)  Weight ratio ~ 4  Spiking Stability 180 pulses | Convolutional neural network accuracy 84% | No | ^[^[^4^](#_ENREF_4)^]^ |
| **5** | **Cu/FF : PAA Na^+^/Cu**  **(PDMS substrate: highly Flexible and robust)**  **(Flexible)** | **Resistive swiching**  **± 4.5 V** | **+3 V and -3 V**  **(100 pulses for potentiaon and depression)**  **(1ms duty interval)**  **Weight ratio ~ 3.42**  **Spiking Stability 5400 pulses** | **Convolutional neural network accuracy 85%** | **yes** | **This work** |

1. **Comparison of liquid neuromorphic resistive memory devices.**

**Table. S1.** Comparison of iontronic liquid neuromorphic resistive memory devices.

1. **Comparison of self-powered resistive memory devices.**

| **No** | **Resistive memory Device** | **Operating Voltage and endurance** | **Self-powered integrating unit (TENG or PENG) with memristor** | **Application** | **Ref:** |
| --- | --- | --- | --- | --- | --- |
| **1** | Ag/a-carbon/Ag  (Quartz Plate as substrate) | Bipolar resistive switching  ±2 V and 10^4^ s retention | (TENG- planer mode)  Ag/a-carbon quart/wlwctrargol/Ag  (Quartz plate as substrate) | SET and RESET memristor | ^[^[^5^](#_ENREF_5)^]^ |
| **2** | AZO/Ta_2_O_5_/Au  (Glass Substarte) | Bipolar resistive switching  -1 V to + 2V and 80 cycles | (TENG- single electrode)  Al/PTFE (skin touch)  (Al tape as substrate) | Touch program and electrically erase for  data storage in the memristor array | ^[^[^6^](#_ENREF_6)^]^ |
| **3** | Si/TaO_x_/Ag  (Si substrate) | Threshold switching  0V to 5V and 30 cycles | (TENG- contact separation)  PET/ITO PDMS/MXene/ITO/PET  (PET substrate) | Self-powered sensory neuron array for feature recognition | ^[^[^7^](#_ENREF_7)^]^ |
| **4** | TiN/KNbO_3_/  Conductive fabric  (Si/SiO_2_ substrate) | Bipolar resistive switching  ±2 V and 50 cycles | (PENG)  TiN/KNbO3/  Conductive fabric  (PI/PET substrate) | SET and RESET memristor | **^[^**[**^8^**](#_ENREF_8)**^]^** |
| **5** | TiN/(Na0.5K0.5)NbO_3_/ Conductive fabric  (Si/SiO_2_ substrate) | Bipolar resistive switching  ±1.5 V and 50 cycles | (PENG)  TiN/(Na0.5K0.5)NbO_3_/ Conductive fabric  (PI substrate) | SET and RESET memristor | ^[^[^9^](#_ENREF_9)^]^ |
| **6** | TiN/NKN/Pt  (PI substrate) | Multistate resistive switching  ±1.5 V | (PENG)  TiN/NKN/Pt  (PI substrate) | Neuromorphic computing  (SRDP and STDP) | ^[^[^10^](#_ENREF_10)^]^ |
| **7** | ITO/ZnO/Yb_2_O_3_/Au  (PET Substrate) | Unidirectional multistate resistive switching  ±4 V | (PENG)  ITO/ZnO/Al  (PET substrate) | Neuromorphic computing  (SRDP) | This work |
| **8** | **Cu/FF:PAA Na^+^/Cu**  **(PDMS Substrate)** | **Multistate resistive switching ±4.5 V and 100 cycles** | **TENG/EMG**  **TENG: Al/PTFE/ (spacer)/FF/Al**  **EMG: Magnet and Cu Coil with n= 1200 turns**  **(PDMS and PET Substrate)** | **Neuromorphic computing**  **(SRDP)** | **This work** |

**Table. S2.** Comparison of self-powered resistive memory devices.

1. **CNN simulation architecture used for hardware implementation of the neural network:**

For simulation, we employ CIFAR-10 images as input data, characterized by dimensions of 32x32x3. The output of the Convolutional Neural Network (CNN) applied to this dataset comprises 10 units, where classes are encoded into one-hot vectors. Error updating is facilitated through the chain rule, comparing the one-hot vector representing the ground truth label with the output of the simulation. We aim to utilize CNN simulation within a memristor array, leveraging the conductance of the device as weights. We elaborate on the simulation's support, particularly within the Neuro Sim 2.0 simulator, which offers advanced capabilities for modelling neural networks. This simulator provides a comprehensive environment for conducting experiments with neural architectures, offering features such as customizable neuron models, synaptic plasticity mechanisms, and parallel computing support. With Neuro Sim 2.0, researchers can explore the dynamics of complex neural networks, investigate the effects of various parameters on network behaviour, and simulate large-scale brain-inspired systems efficiently. Returning to the specifics of CNN operation within the memristor array, the conventional mapping for the convolutional layer is illustrated in Figure S1. Here, kernels are transformed into elongated columns, facilitating efficient computation within the memristor array architecture. Each kernel traverses the input data, performing element-wise multiplications with a prescribed stride and aggregating partial sums to generate outputs. Notably, certain input data may be reused in computations, optimizing resource utilization. As a consequence, each element of the output feature map (OFM) is derived from the accumulation of dot products with its corresponding kernel. This iterative process continues until all kernels have been processed, resulting in the generation of output feature maps, as depicted in Figure 2h of the main manuscript. Through this methodology, the CNN simulation within the memristor array achieves efficient and scalable computation, promising advancements in neuromorphic computing paradigms.


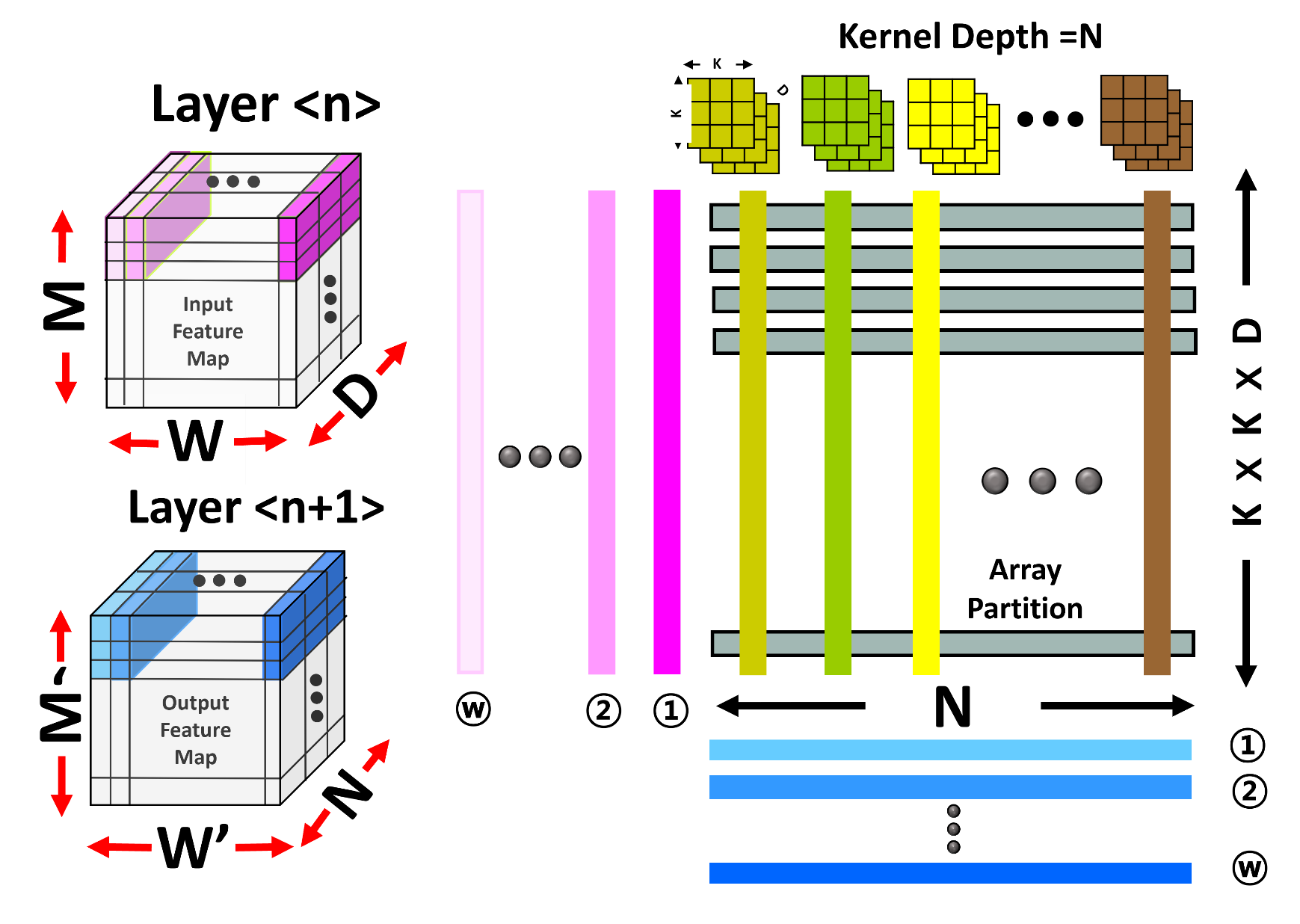


**Figure S1.** The example of processing convolution with input and weight data.

Further, the convolution neural network (CNN) can be implemented in a hardware manner as details are provided as follows: Further, the convolution neural network (CNN) can be implemented in a hardware manner as given in Figure S2 showing the computing operation architecture based on analogue eNVM 1T1R synaptic array with sequential readout. Also, this figure shows how to treat cell arrays. The Word line (WL) can be considered as a switch, which controls the transistor. The source line (SL) is connected transistor source and the bit line (BL) is connected to the top electrode of the eNVM cell. The bottom electrode is connected to the drain of the transistor. In this method, this array can’t perform parallel weight sum. So, BL is horizontal to WL as shown in Figure S2. The BL receives an input voltage, which results in SL being able to read out the weighted sum current. The switch matrix is connected to all BL and transmission gate control signals are stored in registers. During the sum operation, the input signal is loaded, and BLs connected to the input voltage or ground. The role of the WL/BL decoder make all transistors transparent for weighted sum. A multiplexer (MUX) is a connected column line in the synaptic array. Mux shares the read periphery circuits with the column line in the memristor array. Resistive memory devices are used as embedded nonvolatile memory (eNVM). When input comes in eNVM, output will be current. These eNVM arrays perform the CNN algorithm as shown in Fig.S1. Adder and register are integrated into all weight sums. At the bottom of the synaptic core the adder and shift register shift and add all weighted sum results at each input vector bit cycle to get the final weighted sum^[^[^11^](#_ENREF_11)^]^.


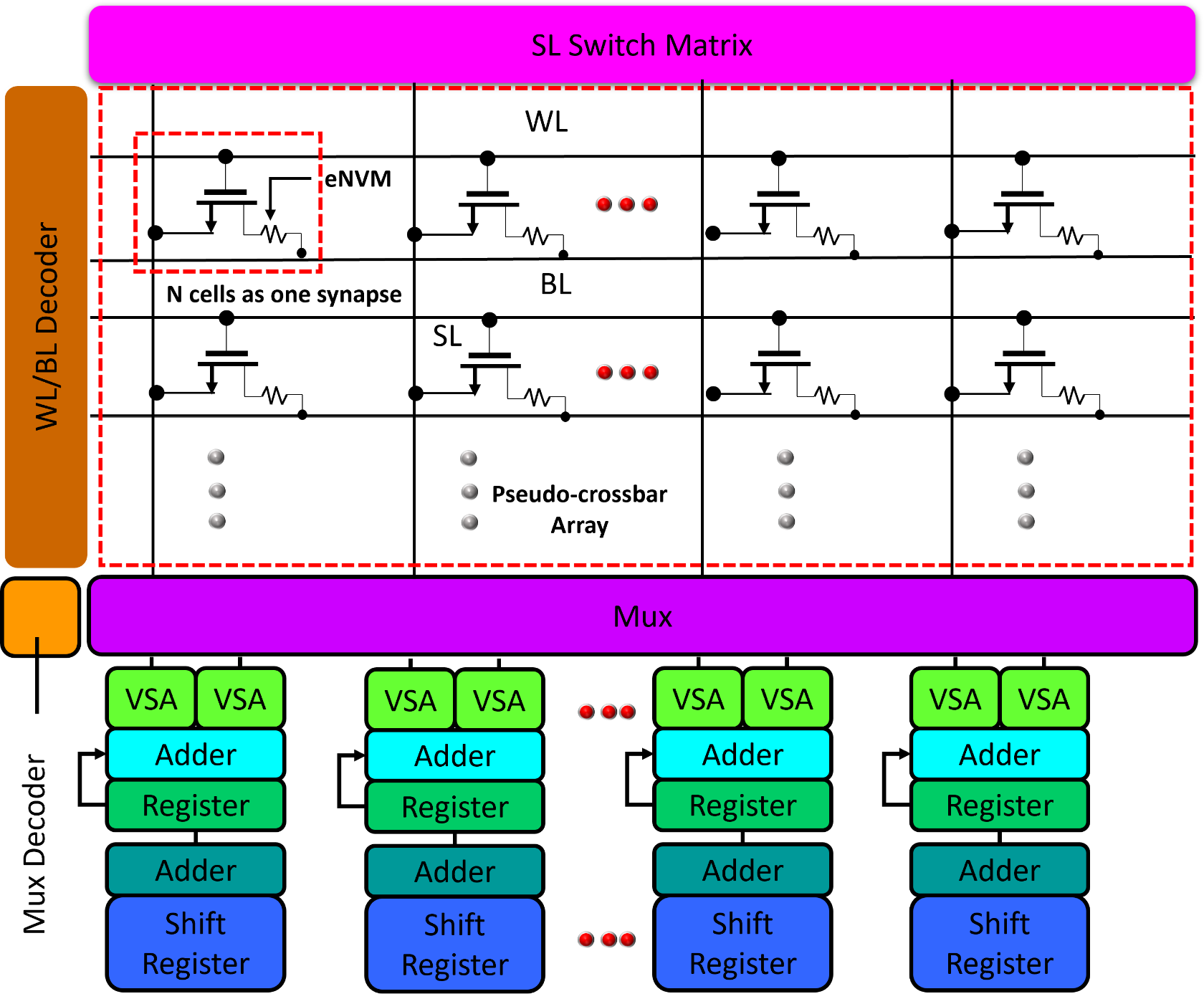


**Figure S2.** Analog eNVM ITR synaptic array with sequential read out.

In neural network, each neuron layer is connected to every neuron of next layer, each connections represents weight. The weight can be updated using a memristor crossbar array to make a neural network. So, weight can be replaced by conductance G_i_. Therefore, vector matrix multiplication can be operated this crossbar array by Ohm’s law and Kirchhoff’s law efficiently. The matrix function can be expressed as follow:

$\left[ \begin{matrix} I_{1} \\ \vdots\\ I_{N} \end{matrix} \right]=\left[ \begin{matrix} G_{11} & \cdots& G_{1N} \\ \vdots& \ddots& \vdots\\ G_{N1} & \cdots& G_{NN} \end{matrix} \right] \left[ \begin{matrix} V_{1} \\ \vdots\\ V_{N} \end{matrix} \right] (1$)

Where $V_{n}$ is input voltages, $I_{n}$ is output current and $G_{ij}$ is conductance. Therefore, output $I_{n}$ can be collected from each bit line. In detail process for training, it has two main steps, which is the feed forward and back propagation process. The role of feed forward process is utilized to obtain error between the output result and true value. In back propagation process, the weights are updated based on feed forward result. Update through back propagation process is repeated iteration process until error is eliminated. Especially, if update for back propagation process, the nonlinear weight is used according to conductance of memristor.

1. **Mechanism study and charge transfer study of Ferrofluid TENG:**

**
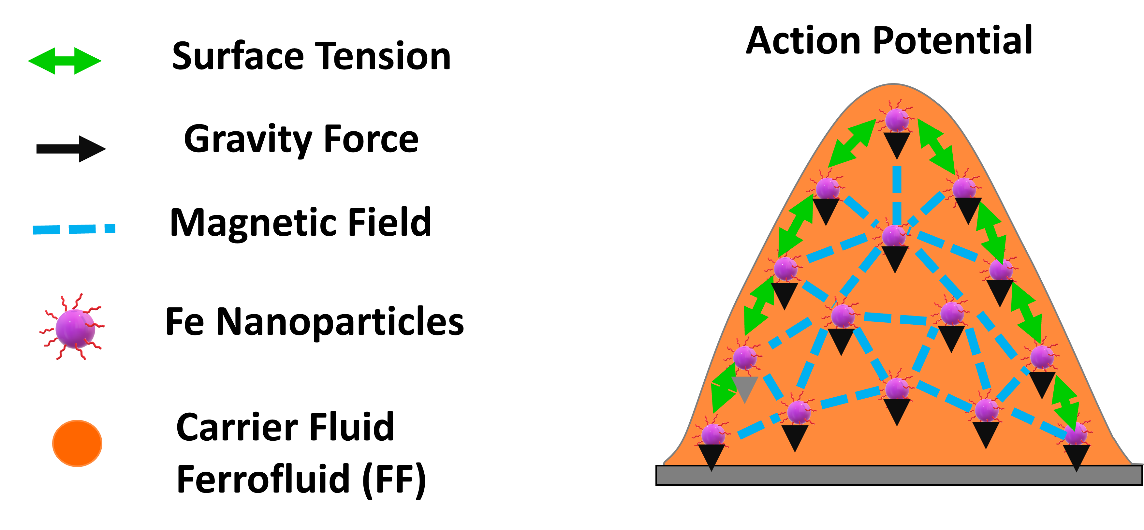
**

**Figure S3.**  Force analysis of FF under magnetic field.

**
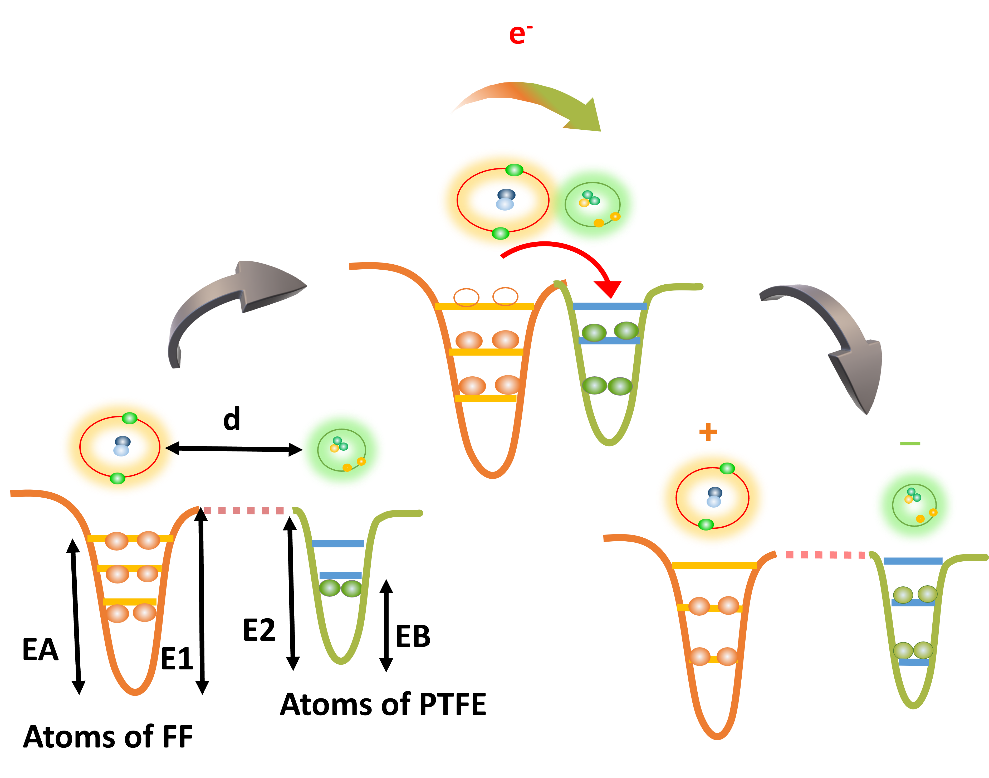
**

**Figure S4.**  Explanation of contact electrifcation phenomenon by electron cloud interaction.

1. **Power performance of FF TENG and EMG:**


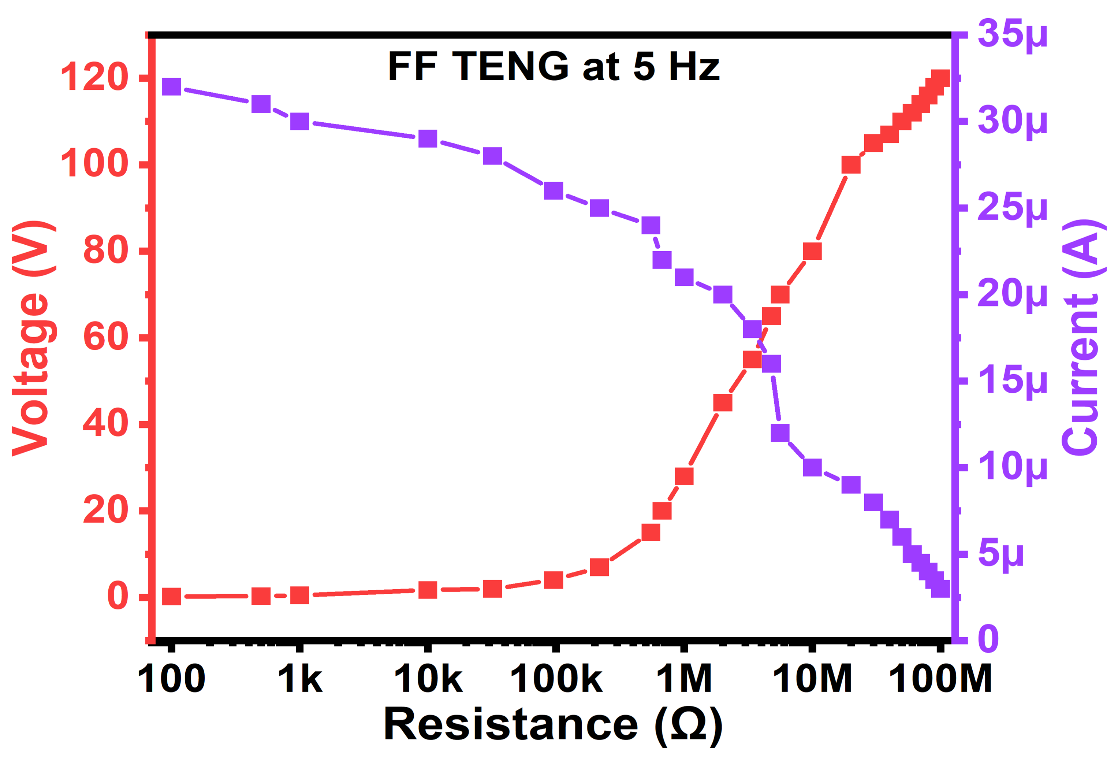


**Figure S5.** The loading resistance effect on current and voltage of FF TENG at 5 Hz**.**


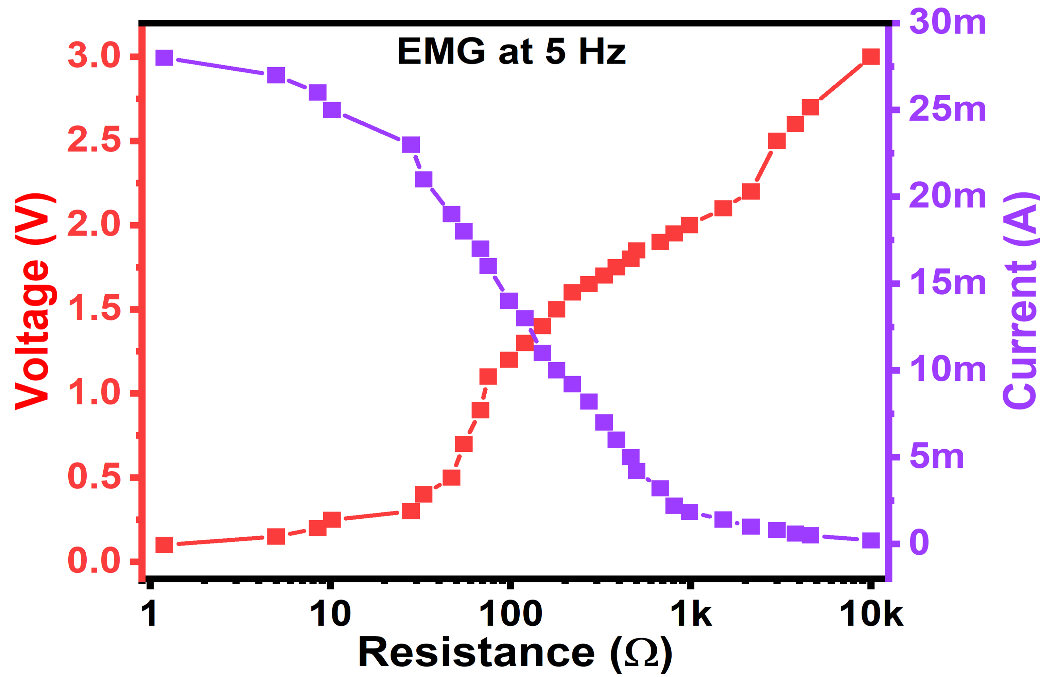


**Figure S6.** The loading resistance effect on current and voltage of EMG at 5 Hz**.**

1. **Stability of FF TENG/EMG Device**

**
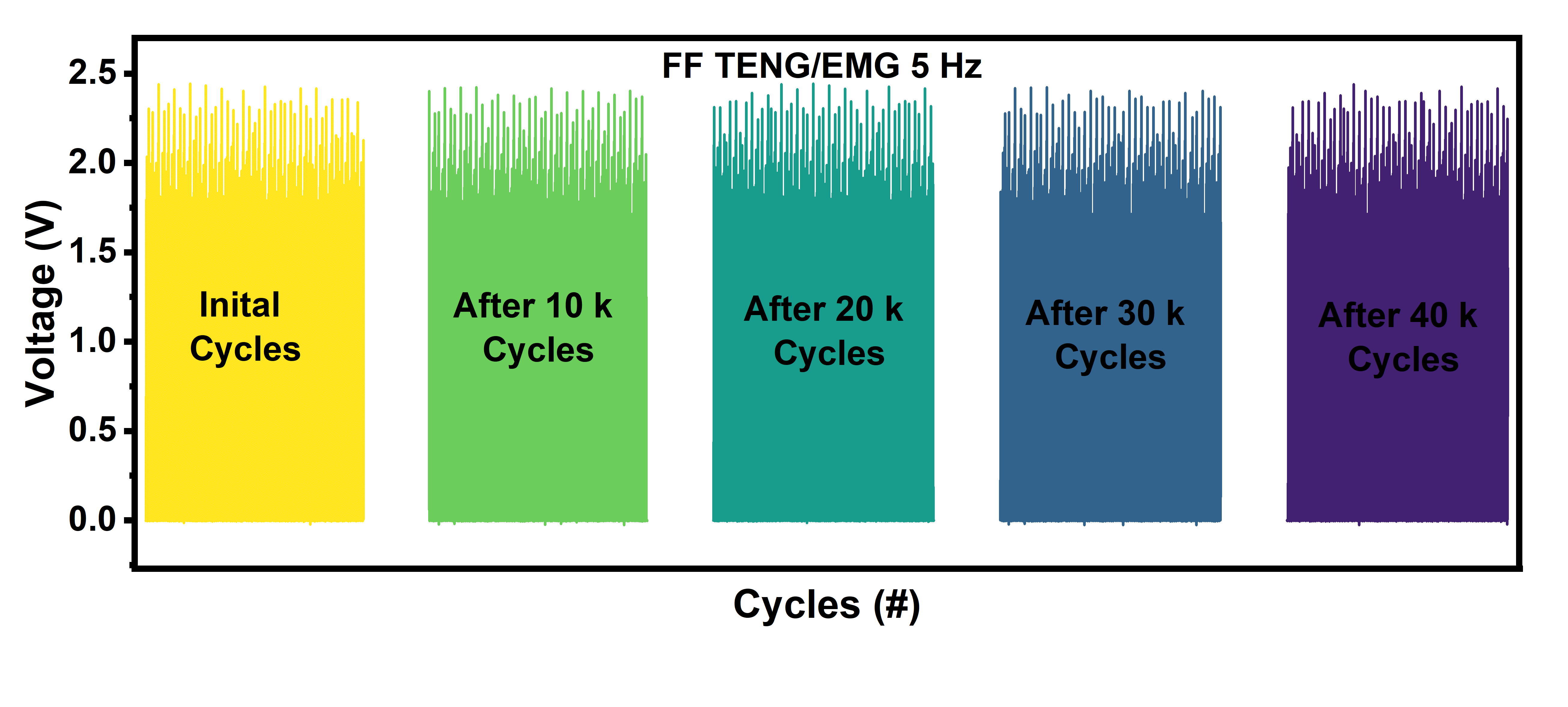
**

**Figure S7.** The stability of FF TENG/EMG for 40,000 cycles at 5 Hz**.**

**Reference**

[1] P. Zhang, M. Xia, F. Zhuge, Y. Zhou, Z. Wang, B. Dong, Y. Fu, K. Yang, Y. Li, Y. He, R. H. Scheicher, X. S. Miao, *Nano Letters* **2019**, *19*, 4279-4286. <https://doi.org/10.1021/acs.nanolett.9b00525>.

[2] M. U. Khan, Q. M. Saqib, M. Y. Chougale, R. A. Shaukat, J. Kim, J. Bae, *Microsystems & Nanoengineering* **2021**, *7*, 78. <https://doi.org/10.1038/s41378-021-00305-7>.

[3] M. Y. Chougale, M. U. Khan, J. Kim, R. A. Shaukat, Q. M. Saqib, S. R. Patil, T. D. Dongale, A. Bermak, B. Mohammad, J. Bae, *Advanced Engineering Materials* **2022**, *24*, 2200314. <https://doi.org/https://doi.org/10.1002/adem.202200314>.

[4] M. U. Khan, J. Kim, M. Y. Chougale, C. M. Furqan, Q. M. Saqib, R. A. Shaukat, N. P. Kobayashi, B. Mohammad, J. Bae, H.-S. Kwok, *Microsystems & Nanoengineering* **2022**, *8*, 56. <https://doi.org/10.1038/s41378-022-00390-2>.

[5] G. Zhou, Z. Ren, L. Wang, J. Wu, B. Sun, A. Zhou, G. Zhang, S. Zheng, S. Duan, Q. Song, *Nano Energy* **2019**, *63*, 103793. <https://doi.org/https://doi.org/10.1016/j.nanoen.2019.05.079>.

[6] Y. Sun, X. Zheng, X. Yan, Q. Liao, S. Liu, G. Zhang, Y. Li, Y. Zhang, *ACS Applied Materials & Interfaces* **2017**, *9*, 43822-43829. <https://doi.org/10.1021/acsami.7b15269>.

[7] W. Ye, J. Lin, X. Zhang, Q. Lian, Y. Liu, H. Wang, S. Wu, H. Chen, T. Guo, *Nano Energy* **2022**, *100*, 107525. <https://doi.org/https://doi.org/10.1016/j.nanoen.2022.107525>.

[8] T.-H. Lee, H.-G. Hwang, S. Jang, G. Wang, S. Han, D.-H. Kim, C.-Y. Kang, S. Nahm, *ACS Applied Materials & Interfaces* **2017**, *9*, 43220-43229. <https://doi.org/10.1021/acsami.7b11519>.

[9] B.-Y. Kim, W.-H. Lee, H.-G. Hwang, D.-H. Kim, J.-H. Kim, S.-H. Lee, S. Nahm, *Advanced Functional Materials* **2016**, *26*, 5211-5221. <https://doi.org/https://doi.org/10.1002/adfm.201505569>.

[10] B.-Y. Kim, H.-G. Hwang, J.-U. Woo, W.-H. Lee, T.-H. Lee, C.-Y. Kang, S. Nahm, *NPG Asia Materials* **2017**, *9*, e381-e381. <https://doi.org/10.1038/am.2017.64>.

[11] P. Chen, X. Peng, S. Yu, *IEEE Transactions on Computer-Aided Design of Integrated Circuits and Systems* **2018**, *37*, 3067-3080. <https://doi.org/10.1109/TCAD.2018.2789723>.
